# *Clonorchis sinensis* secretory protein CsAg17 vaccine induces immune protection

**DOI:** 10.1186/s13071-020-04083-5

**Published:** 2020-04-25

**Authors:** Xuelian Bai, Jin-Ho Song, Fuhong Dai, Ji-Yun Lee, Sung-Jong Hong

**Affiliations:** 1 Department of Medical Environmental Biology, Chung-Ang University College of Medicine, Seoul, Republic of Korea; 2 Clinical Medicine Laboratory, Binzhou Medical University Hospital, Binzhou, Shandong People’s Republic of China; 3 Department of Pharmacology, Chung-Ang University College of Medicine, Seoul, Republic of Korea; 4 Department of Parasitology, School of Biology and Basic Medical Sciences, Medical College, Soochow University, Suzhou, Jiangsu People’s Republic of China

**Keywords:** *Clonorchis sinensis*, Clonorchiasis, Liver fluke, Vaccine, cDNA vaccine, CsAg17, Worm burden, T cell, Cytokine

## Abstract

**Background:**

Clonorchiasis is endemic in East and Southeast Asian countries. For a preventive strategy against infectious diseases, vaccination is the most effective. Here, we evaluated the molecular characteristics and immune responses of CsAg17 protein from *Clonorchis sinensis*, and investigated its protective effects against *C. sinensis* challenge.

**Methods:**

A cDNA clone encoding CsAg17 protein and containing a secretory signal peptide at the N-terminus was retrieved from the *C. sinensis* transcriptome bank. Recombinant CsAg17 B-cell epitope protein and cDNA vaccines were produced and their immune responses were evaluated in FVB mice. The proportional changes of CD3^+^/CD4^+^ and CD3^+^/CD8^+^ T cells were detected by flow cytometry, and immune effectors were measured by ELISA.

**Results:**

The *CsAg17* mRNA was transcribed at a higher level in *C. sinensis* adults than in metacercariae. The CsAg17 protein was distributed in the sperms, oral and ventral suckers, and mesenchymal tissues of *C. sinensis* adults. In mice challenged with *C. sinensis* metacercariae, vaccination with CsAg17 protein and cDNA resulted in a reduction to 64% and 69% in worm burden, respectively. Both CsAg17 protein and cDNA vaccines increased the proportion of CD3^+^/CD4^+^ and CD3^+^/CD8^+^ T cells and stimulated the production of Th1 type cytokines such as interleukin (IL)-2, IL-12, and interferon-γ, while maintaining minimum levels of Th2 cytokines. The levels of IgG specific to CsAg17 protein steeply increased in the two vaccinated groups from 2 weeks after immunization. The liver tissue retained good morphology in the mice vaccinated with CsAg17 protein or cDNA, whereas severe inflammation and large serous cysts were observed in the liver of the unvaccinated mice.

**Conclusions:**

Vaccination with CsAg17 protein and cDNA reduced the pathological changes in the bile duct and liver, and ameliorated the worm burden *via* cellular and humoral immune responses. Thus, they may serve as good vaccine candidates against *C. sinensis* infections.
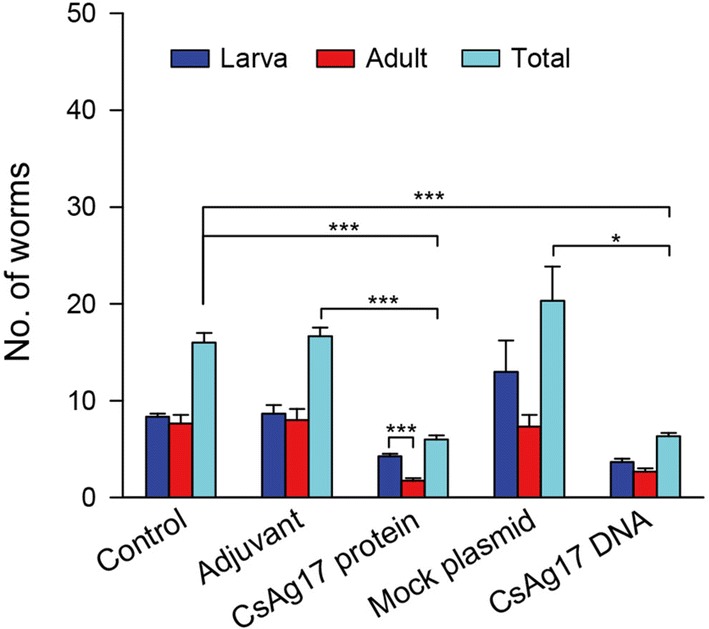

## Background

Clonorchiasis is an endemic common in Asian countries such as China, Korea, and Vietnam. In this region, an estimated 35 million people were infected with *Clonorchis sinensis* [1]. Mammals are the definitive hosts, and humans acquire infection from eating raw or undercooked freshwater fish, the second intermediate hosts and the carriers of metacercariae. After ingestion, the metacercariae excyst in the duodenum, and the newly excysted juvenile flukes migrate up with bile chemotaxis into the bile duct through the ampulla of Vater. The juveniles then grow into adults in the intrahepatic bile duct [2].

Infection with *C. sinensis* may cause serious pathological changes in the bile duct, including a marked dilatation of the duct, thickening of the ductal wall, periductal inflammation, and hyperplasia of the biliary mucosa. *Clonorchis sinensis* has been classified as a biological carcinogen by the International Agency for Research on Cancer, given its association with cholangiocarcinoma [3]. Pathological changes such as periductal fibrosis and cellular infiltration, especially during chronic infection, may take a long time for abatement after deworming.

Vaccination is an effective measure to prevent human infections against pathogens. Several generations of vaccines such as live, attenuated, and subunit vaccines are available. Protein vaccines offer the advantage of inducing a quick immune response but may be unstable and induce only a limited effect. As third-generation vaccines, DNA vaccines engineered to carry DNA fragments encoding antigenic proteins, often generate superior protective antibodies. DNA vaccines present both major histocompatibility complex (MHC) class I and II molecules, which polarize T helper cells towards type 1 (Th1) or type 2 (Th2) [4] and provide a long-term response to immunogens [5].

Humoral and cellular immunities are crucial for mediating protection against *C. sinensis* infection of the bile duct. Secretory proteins are more commonly presented to the host immune system to provoke immune responses. Several vaccine candidates have been proposed against *C. sinensis* infection [6–8], with protective effects in terms of reduction in worm burden ranging between 32–54%. Mice immunized with *Bacillus subtilis* spore displaying *C. sinensis* paramyosin revealed a 48–51% reduction in parasite egg burden [9]. However, the protective efficacy against *C. sinensis* infection in the form of vaccines is yet to be exploited further. It is, therefore, imperative to develop improved vaccine candidates that may induce stronger immune responses and exhibit higher protective efficiencies against *C. sinensis* infections.

The antigenic protein CsAg17 was selected from the secretory proteins of *C. sinensis* [10]. CsAg17 protein was suggested to provoke protective immune responses in mammalian hosts against *C. sinensis* infection. We here elucidated the immune protective potential of CsAg17 protein and cDNA vaccines against *C. sinensis* infection.

## Methods

### DNA sequencing and structure prediction

**Fig. 1:**
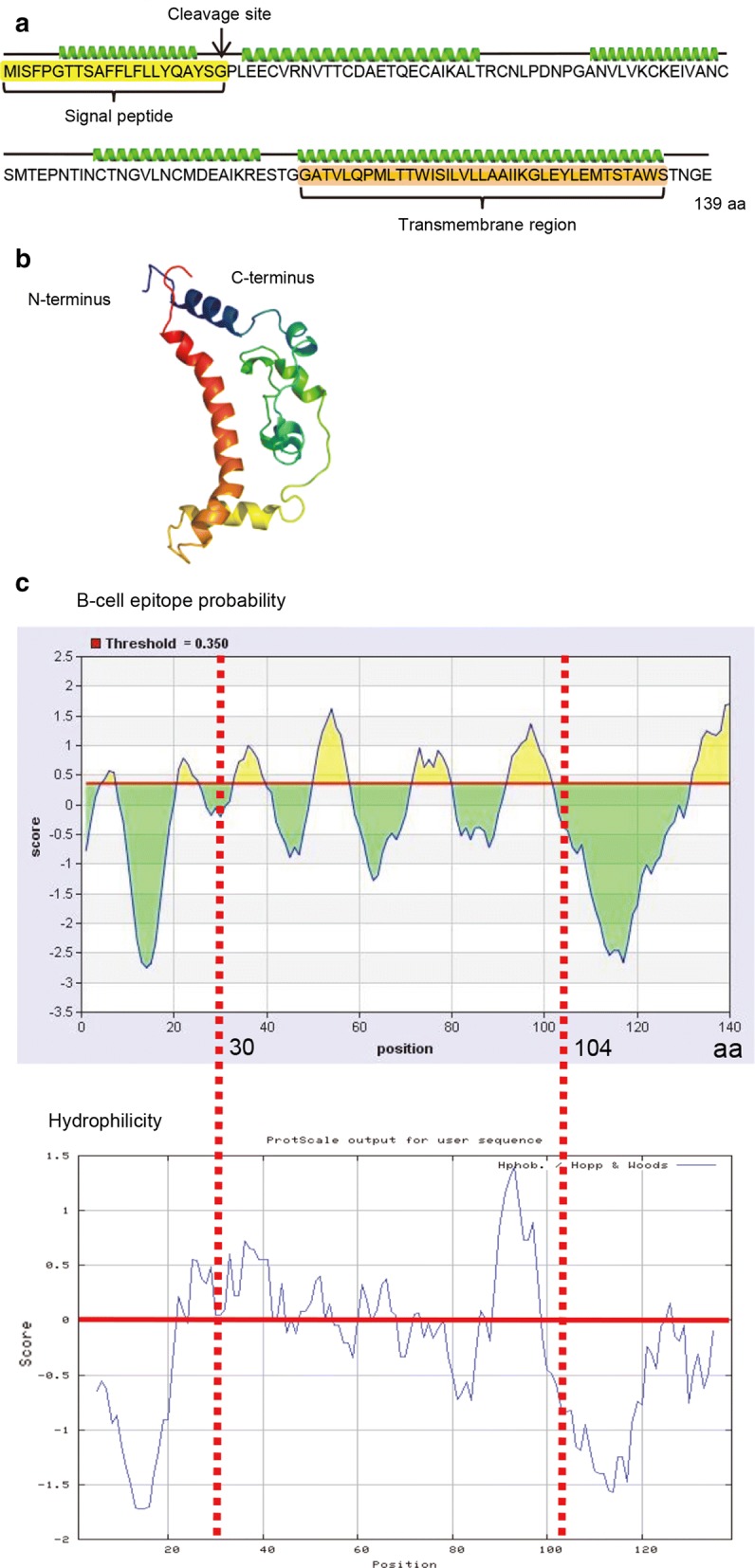
Deduced structure of *C. sinensis* CsAg17 polypeptide. **a** Secondary structure. The signal peptide is shown in yellow frame. The arrow indicates the cleavage site of the signal peptide Alpha-helices are shown in green spirals. **b** Tertiary structure of CsAg17. **c** Prediction of B cell epitope and hydrophilicity. The protein fragment with high antigenicity (yellow) and hydrophilicity was selected to produce a soluble protein

An expressed sequence tag (EST) encoding CsAg17 polypeptide (ID number: CSA19133-3 (CS-N-50-4a-T3_F10)) was retrieved from the *C. sinensis* EST library database at the Korea National Institute of Health and its clone from the *C. sinensis* transcriptome glycerol stock [10, 11]. This cDNA clone was sequenced in full stretch to reach poly(A) tail. The putative polypeptide sequence was predicted using the ExPASy translation tool [12]. BLASTx was used for a CsAg17 homologue search in the National Center for Biotechnology Information (NCBI) database. Functional domains of CsAg17 polypeptide were predicted using the TMHMM Server, v. 2.0. The secondary and tertiary structures of the CsAg17 polypeptide were predicted using Phyre2 [13]. To produce the soluble form of protein with acceptable antigenicity, the putative peptide sequence of CsAg17 was analyzed for the B cell epitope using the IEDB program [14]. From the output, a fragment of high antigenicity and hydrophilicity (30–104th amino acid) was selected (Fig. 1). This fragment was predicted by Phyre2 to have 43% homology to merozoite surface protein 1 (MSP1).

### Quantitative analysis of *CsAg17* mRNA in developmental stages

Total RNA was extracted from the *C. sinensis* adults and metacercariae using TRIzol reagent (Ambion, Foster City, CA). The first-strand cDNA was synthesized with oligo-d(T) primer and Power cDNA Synthesis kit (iNtRON Biotechnology, Gyeonggi-do, Korea) according to the manufacturer’s instruction. The level of *CsAg17* mRNA transcription was measured by real-time quantitative polymerase chain reaction (RT-qPCR). Specific forward (5′-CGT CAG CCT TCT TTC TCT T-3′) and reverse (5′-TCT TGG GTT TCT GCG TCA CA-3′) primers were designed using the Oligo 6.71 program (Molecular Biology Insights Inc., Cascade, WA, USA). The genes of β-actin, calcyphosin, and phosphoglycerate kinase were reported to be expressed stably in both *C. sinensis* adults and metacercariae, and thus, employed as reference for relative quantification of the target gene expression [15]. The reaction mixture (10 μl) comprised 70 ng of *C. sinensis* cDNA, each primer pair of CsAg17 or reference genes, and 1 μl of 10× master mix (FastStart SYBR Green I Kit, Roche, Mannheim, Germany). Thermal cycling was performed using a LightCycler 1.5 (Roche) as follows: the reaction mixture was heated to 95 °C for 15 min and subjected to 45 cycles of 95 °C for 10 s, 60 °C for 10 s, and 72 °C for 30 s. A melting curve was plotted by heating the PCR products to 95 °C for 10 s and then cooling to 65 °C at a rate of 0.1 °C/s. Data were analyzed using the LightCycler program. Relative transcription level was calculated using the 2^−ΔΔCq^ method [16].

### Production of recombinant CsAg17 B cell epitope protein

The full-length CsAg17 recombinant protein was obtained in an insoluble form upon expression as a fusion protein to His-tag peptide in *Escherichia coli* BL21 (DE3). The protein was purified under denaturation conditions using sarcosyl or urea. After dialysis against phosphate-buffered saline (PBS), the majority of the recombinant CsAg17 protein was retained as an aggregate. Thus, a soluble fragment of high antigenicity and hydrophilicity (30–104th amino acid) was selected and the corresponding coding cDNA region was amplified by PCR using the forward (5’-GCA GAG AAT TCG AGA AAC GTA ACA TGT GAC GCA-3’) and reverse (5’-GTA GTC TCG AGT TGA AGT ACG GTT GCA CCA C-3’) primers extended with *EcoR*I or *Xho*I restriction enzyme site (underlined). Amplicons were purified, double-digested with restriction enzymes *EcoR*I and *Xho*I, and subcloned into an expression vector, pET-23b(+). After transforming *E. coli* BL21 (DE3) pLysS with this plasmid, the expression of the recombinant protein was induced with 0.1 mM isopropyl β-d-1-thiogalactopyranoside (IPTG) for 4 h at 37 °C. The cultured *E. coli* cells were harvested and lysed by repeat freezing-thawing (three rounds between liquid nitrogen and 42 °C water bath) and sonication in 1× PBS with 1 s pulse for 10 min (35 vibrations/pulse). After centrifugation at 8000× *rpm* for 30 min at 4 °C, the supernatant was incubated with Ni-NTA agarose (Qiagen, Hilden, Germany) containing 10 mM imidazole at 4 °C for 3 h. The beads were washed twice with Ni-NTA native lysis buffer containing 20 mM imidazole, and the recombinant protein was eluted with Ni-NTA native lysis buffer containing 100 mM imidazole. The recombinant CsAg17 partial protein was dialyzed against 1× PBS and subjected to 12% gradient gel electrophoresis. Polyclonal antibodies were produced by immunizing BALB/c mice with the recombinant CsAg17 partial protein and used for immunohistochemical staining of *C. sinensis* adults [17].

### Construction of CsAg17 cDNA vaccine

Full-length CsAg17 cDNA was amplified with PCR using the specific forward (5′-GAG ATA AGC TTG CCA CCA TGA TTT CGT TTC CTG GC-3′) and reverse (5′-CTA TCG GAT CCT TAT TCA CCA TTC GTA CTC CAT G-3′) primers carrying the Kozak consensus sequence GCCACC and *Hind*III or *BamH*I restriction enzyme site (underlined). The amplicon was purified and double-digested with restriction enzymes *Hind*III and *BamH*I, and subcloned into the eukaryotic expression plasmid vector, pcDNA3.1(+) (Invitrogen, Carlsbad, CA, USA). Ligation was confirmed by restriction enzyme digestion and sequencing. The recombinant plasmid pcDNA3.1-CsAg17 was transformed into *E. coli* DH5α and purified using endotoxin-free plasmid Maxi kit (Qiagen) according to the manufacturer’s instruction. The endotoxin contained was < 0.1 EU/µg plasmid DNA.

### Immunization with recombinant CsAg17 partial protein and DNA vaccines

Seventy FVB mice (female, 7-week-old) were purchased from Samtako Bio Korea Inc. (Osan, Korea) and divided into five groups as follows: CsAg17 protein vaccine group (*n *= 15); Freund’s adjuvant group (*n *= 15); pcDNA3.1-CsAg17 DNA vaccine group (*n *= 15); mock plasmid DNA group (*n *= 15); and blank control group (*n *= 10). For the CsAg17 protein vaccine and Freund’s adjuvant groups, recombinant CsAg17 partial protein (50 μg/mouse) or 1× PBS was emulsified with Freund’s complete adjuvant and intraperitoneally injected into mice. Then the mice were intraperitoneally injected with a booster dose of 50 μg of the recombinant partial protein emulsified with Freud’s incomplete adjuvant or Freud’s incomplete adjuvant alone twice at an interval of 2 weeks.

The mice from the pcDNA3.1-CsAg17 DNA vaccine and mock plasmid DNA groups were injected with 100 μg of pcDNA3.1-CsAg17 DNA or pcDNA3.1(+) into the quadriceps muscles thrice at an interval of 2 weeks. Mice from the blank control group were not subjected to any procedure.

Blood samples were obtained from the tail at pre-immunization, 2 weeks after the first, second, and third immunization, and 4 weeks after infection.

### Infection challenge and worm recovery

Two weeks after the final immunization, 50 *C. sinensis* metacercariae were orally administered to each mouse in all groups using a gavage needle [18, 19]. After 4 weeks, 8 mice in each experimental group and 4 mice from the blank control group were sacrificed and the livers were resected. Each liver was crumbled by hand in saline, and *C. sinensis* adult flukes were collected under a dissecting microscope. Worm reduction rate was calculated as a percentage by comparing the number of worms recovered from the vaccinated and control groups.

### Enzyme-linked immunosorbent assay (ELISA) for anti-CsAg17-specific IgG antibody

Mouse blood samples were collected at 0, 2, 4 and 6 weeks after the first vaccination and 4 weeks after infection. Sera were isolated and used to determine antibody titers with ELISA. Recombinant CsAg17 partial protein was diluted in carbonate buffer at a concentration of 2 μg/ml. The antigen was aliquoted into a 96-well plate (200 μl/well) and incubated at 4 °C overnight. After washing five times with PBS containing 0.1% Tween-20, the plate was incubated with mouse sera (1:6400) for 1 h at 37 °C. A secondary antibody [peroxidase-conjugated goat anti-mouse IgG antibody (1:50,000)]; Sigma-Aldrich, St. Louis, MO, USA) was added to the plate and incubated at 37 °C for 1 h. The samples were treated with the substrate *o*-phenylenediamine and the reaction was terminated with the addition of 8 N sulfuric acid (H_2_SO_4_). Optical density was measured at 490 nm using a microplate reader and all measurements were carried out in triplicate.

### Flow cytometry assay for CD4^+^ and CD8^+^ T cells

The splenocytes (10^6^ cells) were isolated from 3 mice from each group before and after challenge infection, washed twice with PBS (pH 7.4), and resuspended in PBS containing 10% fetal bovine serum (FBS). The splenocytes were incubated with anti-mouse CD16/CD32 antibody and monoclonal antibody of fluorescein isothiocyanate (FITC)-labeled CD4, phycoerythrin (PE)-labeled CD8, and allophycocyanin (APC)-labeled CD3 (eBioscience, San Diego, CA, USA) in a final reaction volume of 20 μl for 30 min on ice in the dark. The cells were washed once with PBS and fixed with 500 μl of 5% formaldehyde. After washing with PBS twice, the cells were resuspended in 1 ml PBS and loaded on a flow cytometry machine (Becton Dickinson, Franklin Lakes, NJ, USA). Cells were selected using the CD3 signal as gate R1 and the proportion of CD4^+^ or CD8^+^ subpopulation in CD3^+^ T cells was analyzed. Data were analyzed using BD CellQuest^TM^ Pro software.

### Splenocyte culture and cytokine assay

Splenocytes from each group were isolated and seeded at a density of 5 × 10^5^ cells/ml in 24-well plates in the presence of recombinant CsAg17 partial protein (12 μg/ml). Concanavalin A (0.5 μg/ml, Sigma-Aldrich) was used as a positive control in all assays. All plates were incubated for 48 h at 37 °C in a 5% CO_2_ humidified atmosphere. Supernatant from the splenocyte culture was collected and incubated at −20 °C. Interleukin (IL)-2, IL-4, IL-10, IL-12 and interferon-γ (IFN-γ) in the supernatant were measured using sandwich ELISA kits (eBioscience). The procedure was as follows: Corning Costar 9018 ELISA plates (Corning Incorporated, NY, USA) were coated with 100 μl/well of IL-2, IL-4, IL-10, IL-12 or IFN-γ capture antibody in 1× coating buffer and incubated overnight at 4 °C. The medium was aspirated, and the plates were washed thrice, followed by blocking with 1× ELISA diluent (200 μl/well) and incubation at 25 °C for 1 h. After washing, 2-fold serial dilutions of standard IL-2, IL-4, IL-10, IL-12 and IFN-γ or splenocyte culture supernatant (100 μl/well) were added to the respective wells and the plates were incubated at 25 °C for 2 h. After washing 3–5 times, detection antibody (100 μl/well) was added to each well and the plate was incubated at 25 °C for 1 h. After washing 3–5 times, the plate was incubated with avidin-horseradish peroxidase (HRP; 100 μl/well) at 25 °C for 30 min. The plate was washed 5–7 times and incubated with 1× TMB solution (100 μl/well) at 25 °C for 15 min, followed by the addition of 50 μl of 2 N H_2_SO_4_. Optical density was measured at a wavelength of 450 nm using a microplate reader (Molecular Devices, Foster City, CA, USA). All assays were performed in triplicate.

### Histopathology of mouse liver

Four weeks after infection, mice were sacrificed, and their livers were excised and observed by naked eye. The livers were fixed in 10% paraformaldehyde, embedded in paraffin, and sectioned into ribbons. The ribbons were deparaffinized, rehydrated, and placed on slides. The slides were stained with a nuclear dye (hematoxylin) and rinsed, followed by counterstaining with eosin. The slides were sequentially rinsed in water, alcohol, and xylene and covered with coverslips. Pathological changes were observed under a light microscope and photomicrographs were obtained.

### Statistical analysis

Data were analyzed using the Student’s t-test and values of *P* < 0.05 were considered statistically significant. All values represent the mean ± standard error (SE) of the mean of more than three independent assays.

## Results

### CsAg17 cDNA and polypeptide

CsAg17 cDNA was 420 bp in length and predicted to encode a polypeptide of 139 amino acid (aa) residues, with an estimated molecular weight of 15.1 kDa. This polypeptide had five helical regions, one of which was the transmembrane domain. A putative secretory signal peptide of 22 aa was predicted with a cleavage site at the N-terminus. The tertiary structure of CsAg17 protein was predicted to have five α-helices and loops (Fig. 1). When blasted against non-redundant database in NCBI, the CsAg17 polypeptide did not reveal any significant homologue, suggesting that it was a unique protein of *C. sinensis* and a good vaccine candidate. The CsAg17 cDNA sequence was deposited in the GenBank database under the accession number MN381946.

### CsAg17 partial protein and immune serum

The recombinant CsAg17 partial protein (Fig. 1) was overexpressed in *E. coli* BL21 (DE3), and remained in the soluble form. As a fusion protein to 6× His tag, CsAg17 was purified using Ni-NTA column under native conditions. The protein appeared as a single band on sodium dodecyl sulfate polyacrylamide gel and had an expected molecular weight of 10 kDa (Additional file 1: Figure S1). This purified recombinant CsAg17 partial protein was used for mouse immunization. Mouse anti-CsAg17 partial protein immune serum specifically reacted with the recombinant and native CsAg17 proteins as well as the recombinant CsAg17 partial protein (Additional file 2: Figure S2).

### Distribution of CsAg17 protein in *C. sinensis* adults

The CsAg17 protein was localized in *C. sinensis* adults with immunohistochemical staining using mouse anti-CsAg17 partial protein immune serum. Strong staining signal was detected in the sperms in the seminal receptacle, ventral sucker, and mesenchymal tissues. Weak signals were observed in the uterine eggs, testes, and vitelline follicles (Additional file 3: Figure S3).

### Transcription level of *CsAg17* during developmental stages

The developmental expression of CsAg17 in *C. sinensis* adults and metacercariae was analyzed with real-time qPCR. Relative transcription level of the target gene *CsAg17* was calculated from Cq (quantification cycle) values of target and reference genes using the 2^−ΔΔCq^ method [16]. As a result, the transcription level of *CsAg17* mRNA was 2.5-fold higher in the adults than in the metacercariae (Additional file 4: Figure S4).

### Antibody production by CsAg17 protein and DNA vaccines

**Fig. 2:**
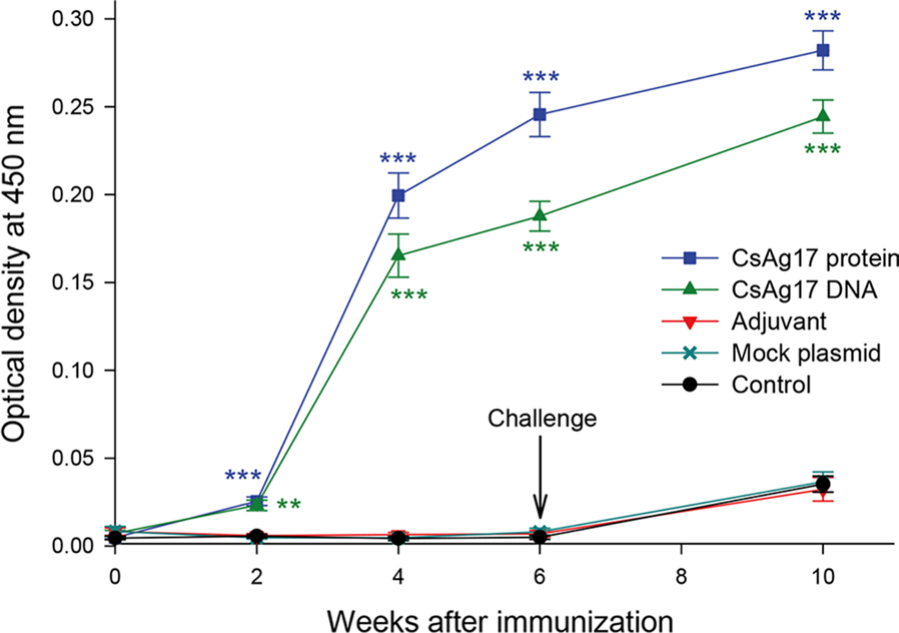
Specific IgG antibody production in mice immunized with CsAg17 vaccines. Mouse serum was diluted 1:6400, and CsAg17-specific IgG level was measured by ELISA. **P < 0.01 and ***P < 0.001 as compared to the blank control

Sera were collected from mice before and after infection challenge and analyzed for the antibodies induced by CsAg17 protein and DNA (Fig. 2). Before infection, CsAg17-specific IgG titer in mice immunized with CsAg17 protein and DNA vaccines steeply increased from 2 weeks after immunization. The IgG titer was higher in the protein vaccinated group than in the DNA vaccinated group. The IgG titer in the mice injected with Freund’s adjuvant or mock pcDNA3.1(+) DNA was parallel with that of the blank control group. After infection with *C. sinensis* metacercariae, the titer of CsAg17-specific IgG antibody continuously increased in both vaccinated groups, but only a slight increase was reported in the three control groups.

### Shift in T cell population

**Fig. 3:**
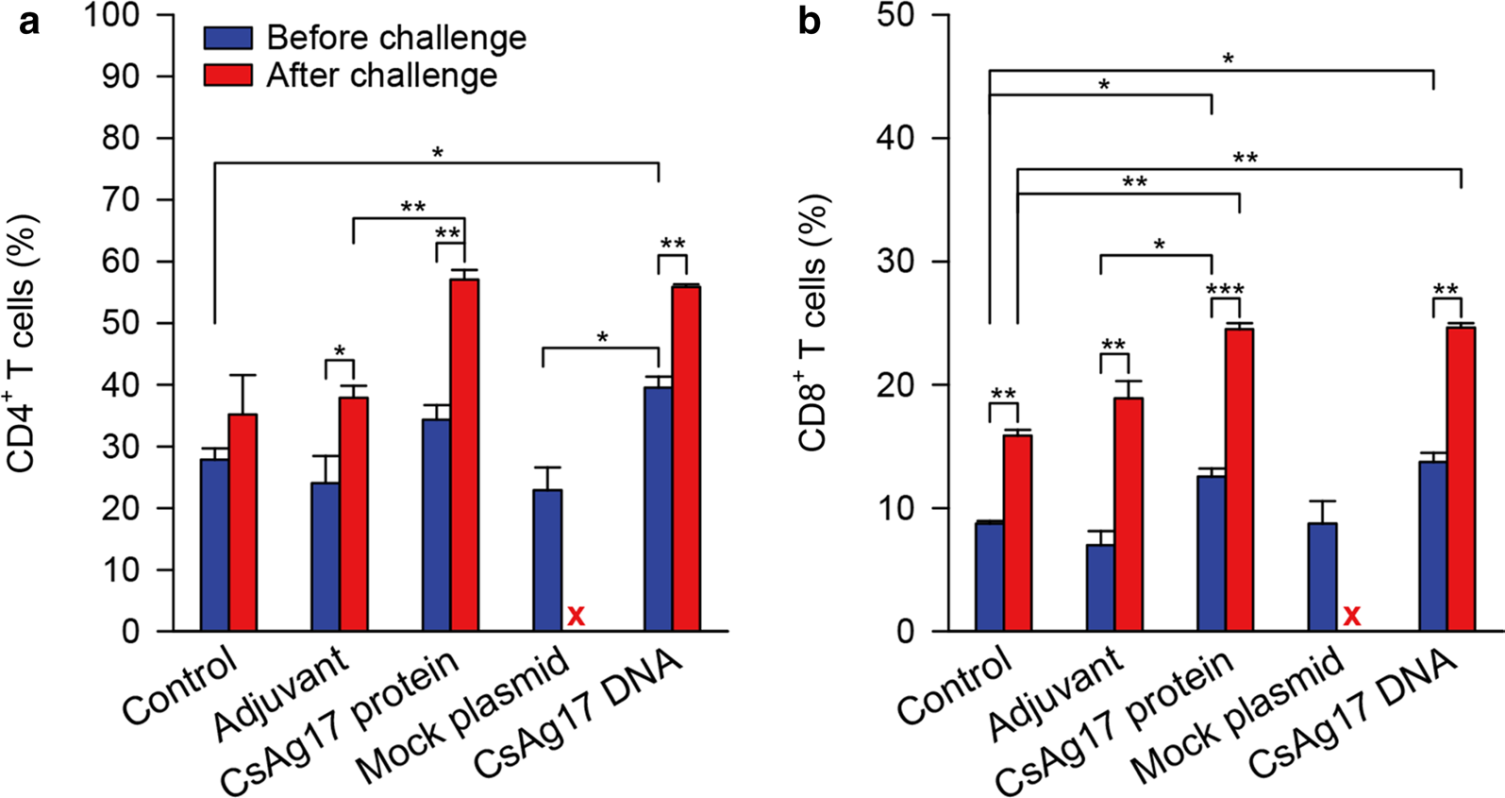
Proportion of CD4^+^ and CD8^+^ T cells in mice immunized with CsAg17 vaccines. Percentage of CD4^+^ (**a**) and CD8^+^ (**b**) T cells. Mouse spleen T cells were sorted by flow cytometry before and after infection. The number of T cells after infection in mice immunized with mock DNA was not large enough for analysis with flow cytometry (x). *P < 0.05, **P < 0.01, ***P < 0.001

The proportion of CD3^+^CD4^+^ and CD3^+^CD8^+^ T cells in the mouse spleen was measured with flow cytometry analysis before and after infection (Fig. 3). Two weeks after the final immunization, the percentages of CD3^+^CD4^+^ and CD3^+^CD8^+^ T cells increased in CsAg17 protein and DNA vaccinated groups. Four weeks after challenge, the percentages of CD3^+^CD4^+^ and CD3^+^CD8^+^ T cells significantly increased in CsAg17 protein and DNA vaccinated groups but were much higher than that observed in the adjuvant, mock and blank control groups. In the adjuvant and blank control groups, a small increase in CD3^+^CD4^+^ T cell population was observed after infection compared to the level detected before infection, whereas the percentage of CD3^+^CD8^+^ T cells significantly increased after the challenge. In the mock DNA group, the size of lymphocytes was much smaller than that of normal lymphocytes and the cell number in gate R1 was not high enough to determine the percentage of CD3^+^CD4^+^ and CD3^+^CD8^+^ T cells.

### Cytokine profile

**Fig. 4:**
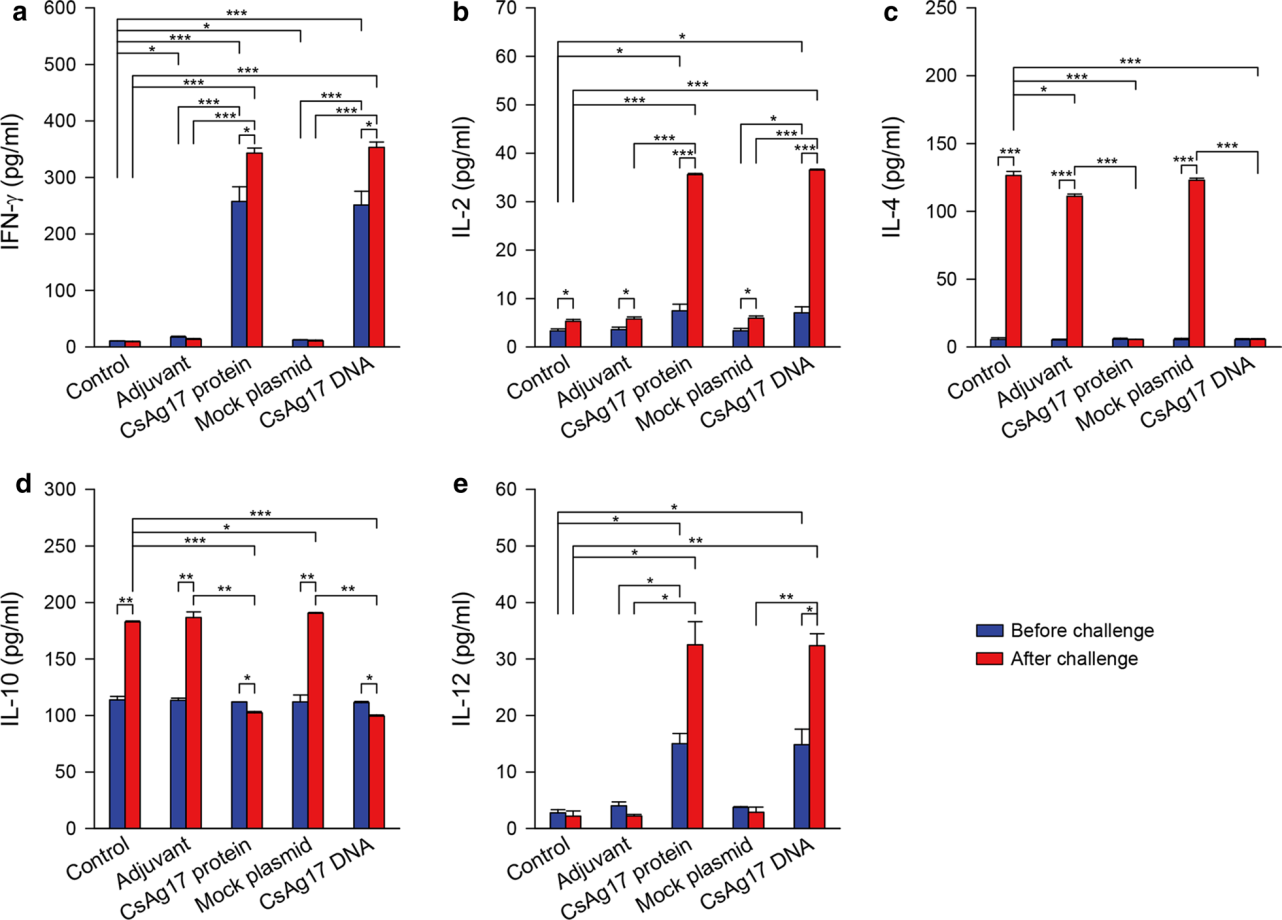
Production of cytokines by splenocytes of mice immunized with CsAg17 vaccines. Mouse splenocytes were isolated and cultured before and after infection. Cytokines IFN-γ (**a**), IL-2 (**b**), IL-4 (**c**), IL-10 (**d**), and IL-12 (**e**) were measured in the supernatant by ELISA. *P < 0.05, **P < 0.01, ***P < 0.001

Mouse spleen lymphocytes were isolated and cultured, and the supernatant was collected for the analysis of cytokine production. Titers of IFN-γ, IL-2, IL-4, IL-10, and IL-12 were measured with ELISA. IFN-γ, IL-2 and IL-12 levels significantly increased in mice vaccinated with CsAg17 protein and DNA 2 weeks after the final immunization and were much high after infection (Fig. 4). In contrast, the levels of these markers in the groups treated with Freund’s adjuvant and pcDNA3.1(+) plasmid were only slightly different from the blank control. The levels of IL-4 and IL-10 were low and similar in all experimental groups at 2 weeks after the final immunization. After infection challenge, however, these profiles showed a tremendous shift. Both IL-4 and IL-10 levels significantly increased in the blank control, Freund’s adjuvant, and mock plasmid treatment groups but reversed in CsAg17 protein and DNA vaccination groups.

### Pathological changes induced by infection challenge

**Fig. 5:**
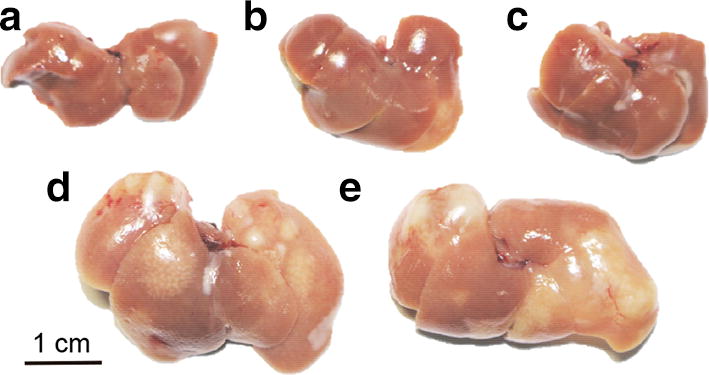
Hepatoprotective effect of CsAg17 vaccines against *C. sinensis* infection. **a** Normal mouse liver. **b**-**e** Livers 4 weeks after infection from mice immunized with CsAg17 DNA vaccine (**b**), CsAg17 protein (**c**), Freund’s adjuvant (**d**), or mock plasmid DNA (**e**)

The livers of mice immunized with CsAg17 protein or DNA vaccines showed mild morphological changes, as evident from swelling and small cysts (Fig. 5). In contrast, the livers from the mice immunized with Freund’s adjuvant or mock DNA showed a marked increase in size accompanied with severe swelling and several large cysts harboring mature mobile *C. sinensis* worms. The swollen liver occupied most of the peritoneal cavity.

**Fig. 6:**
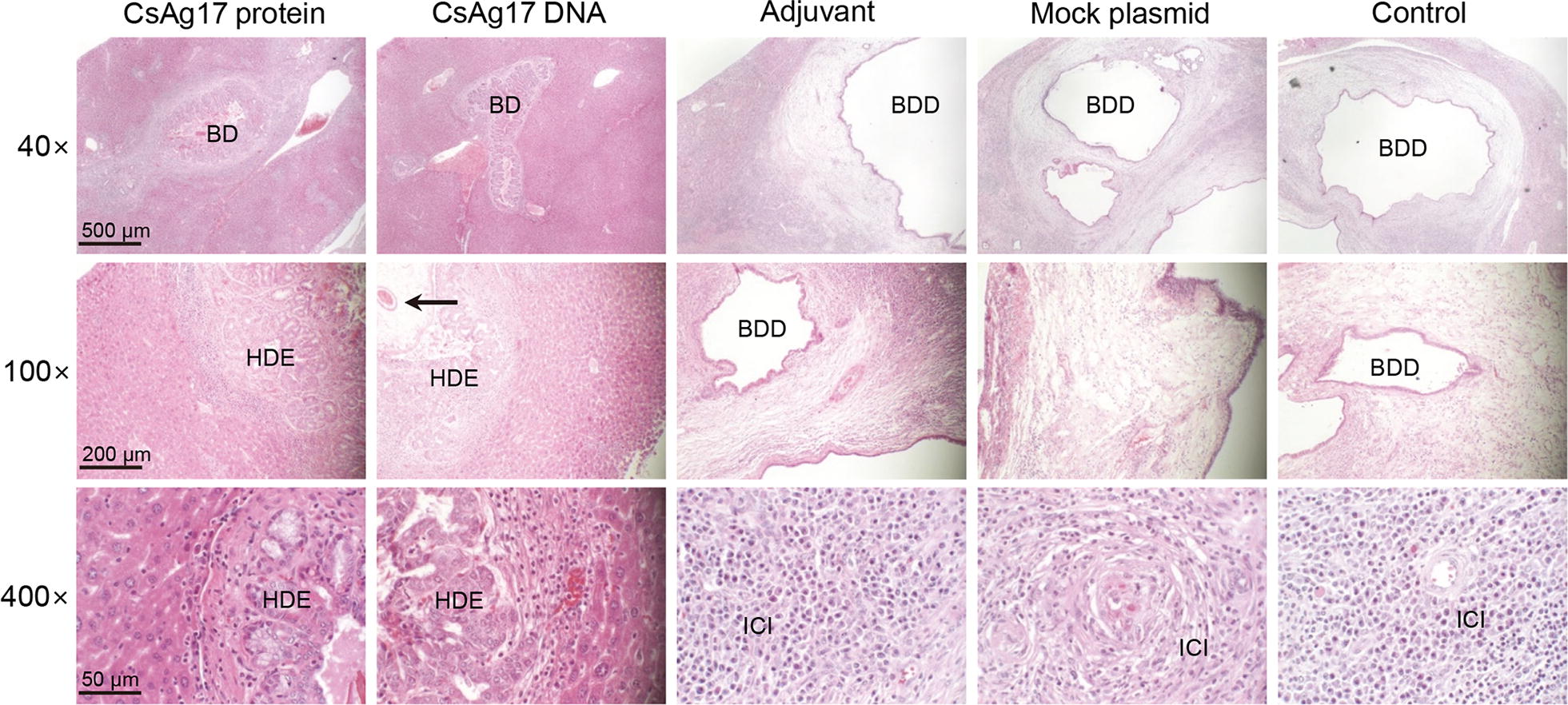
Histopathological changes in the liver infected with *C. sinensis*. Representative images of pathological changes in the liver from mouse infected with *C. sinensis*. The mice were immunized with CsAg17 protein and DNA vaccines, Freund’s adjuvant, mock plasmid DNA, or blank control. An arrow indicates *C. sinensis* worm in the bile duct. *Abbreviations*: BD, bile duct; BDD, bile duct dilatation; HDE, hyperplasia of ductal epithelium; ICI, inflammatory cell infiltration

In the mice vaccinated with CsAg17 protein and DNA, inflammation was mild and a few inflammatory cells were infiltrated around the bile duct (Fig. 6). The inflammation seldom extended into the periductal tissue. Pathological changes in the bile duct included mucosal hyperplasia and moderate periductal fibrosis. In the Freund’s adjuvant and mock DNA groups, dilatation and thickening of the ductal wall was remarkable. Fibrosis was thick and prominent around the bile duct. Inflammation was severe and a dense infiltration of a noticeably large number of eosinophils extending from the bile duct into the periductal hepatic tissue was observed. The hepatocytes showed adipose degeneration and necrosis, which led to hemorrhagic ascites.

### Reduction in worm burden

**Fig. 7:**
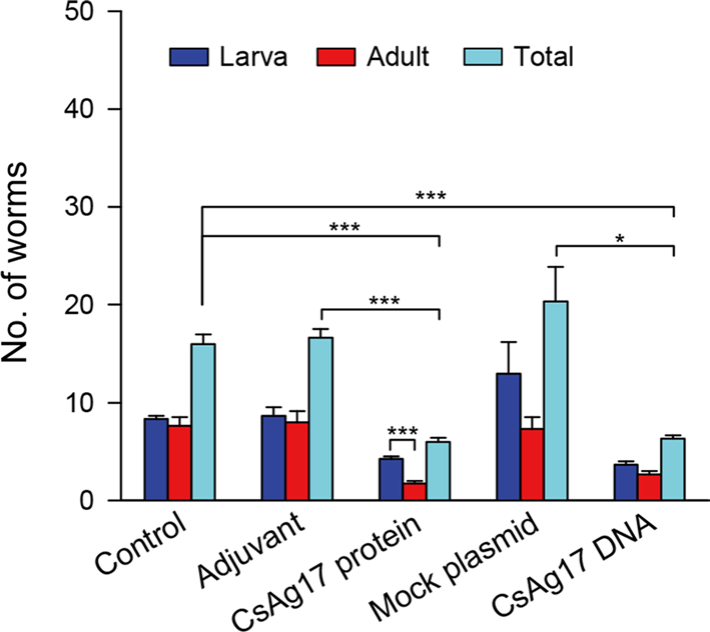
Worm recovery from the liver of mice infected with *C. sinensis*. Mice were immunized with CsAg17 protein and DNA vaccines, Freund’s adjuvant, mock plasmid DNA, or blank control. Two weeks after final immunization, each mouse was orally challenged with 50 *C. sinensis* metacercariae. The adult flukes were recovered from the liver after 4 weeks. *P < 0.05, ***P < 0.001

Worm recovery was significantly lower in the mice from CsAg17 protein and DNA vaccine groups than in that from the Freund’s adjuvant and mock plasmid DNA groups (64% and 69%, respectively, Fig. 7). Moreover, immature worms were predominant in the CsAg17 protein vaccinated group.

## Discussion

Although praziquantel is a good anthelminthic against *C. sinensis* infection, it may not completely eliminate juvenile flukes from the bile duct. Many factors contribute to the global burden of *C. sinensis* infection, one of which is the lack of an effective vaccine. Several studies have been conducted to mediate immune protection against *C. sinensis* infection, thereby leading to some reduction in worm burden. A DNA vaccine encoding a fatty acid-binding protein was able to reduce the worm burden by approximately 30% [20]. In addition, a cysteine proteinase DNA vaccine induced humoral and cellular immune responses and reduced the worm burden by 32% [21].

The distribution of CsAg17 protein in the somatic and reproductive organs is indicative of its role in spermatogenesis and egg production, and highlights the host-parasite interaction. The higher transcription of *CsAg17* in the *C. sinensis* adults than in the metacercariae supports its possible role in adults. Some secretory proteins of *C. sinensis* were shown to induce pathological changes in the infected bile duct and immune responses [20–22]. Thus, CsAg17 protein may permeate into the periductal tissues of the *C. sinensis*-infected liver and stimulate the host immune machinery.

The FVB mouse was susceptible to *C. sinensis* infection and developed serious pathological changes [18]. Vaccination with both CsAg17 protein and DNA induced almost equivalent Th1 and Th2 responses, consistent with the reduction in worm burden in mice challenged with *C. sinensis* metacercariae. DNA vaccines offer several advantages over protein vaccines, including ease of production and stability during storage and shipping. The proteins produced from DNA vaccine *in vivo* are presented as both MHC class I and II molecules [4]. In this direction, the plasmid pcDNA3.1(+) was used as a vector for DNA vaccine construction [23, 24] and proved efficient for the expression of CsAg17 protein in FVB mice.

Humoral immune responses play important roles in the neutralization and elimination of pathogens and toxins. Immunoglobulin G antibody is one of the most important components of the humoral immunity that identifies and neutralizes foreign targets. IgG antibody level increases in an attempt to provide protective immunity against trematode infections. DNA vaccines against *Schistosoma mansoni* and *S. japonicum* elicited high levels of IgG production [25, 26]. A DNA vaccine encoding fatty acid-binding protein stimulated the production of specific IgG2a antibody against *C. sinensis* infection [20]. In the present study, CsAg17 protein and DNA vaccines induced higher production of specific IgG antibody and exhibited protective effects against *C. sinensis* challenge, resulting in worm reduction.

T lymphocytes play a central role in cell-mediated immunity. CD4^+^ T cells activate other immune cells and get activated by antigens presented by MHC II molecules, which are expressed on the surface of antigen-presenting cells [27]. In the FVB mice infected with *C. sinensis*, the decrease in CD4^+^ T cell population could increase their susceptibility to *C. sinensis* infection [18]. Conversely, *C. sinensis* may suppress the proliferation of CD4^+^ T cells. CD8^+^ T cells are cytotoxic and may kill the infective pathogens. These cells are activated by pathogens presented by MHC I molecules. The proportional increase in both CD4^+^ and CD8^+^ T cells in mice immunized with CsAg17 protein and DNA vaccines may contribute to the protective activities against *C. sinensis* infection.

The protective action of CsAg17 vaccines against *C. sinensis* infection was orchestrated with the induction of cytokines, which modulate humoral and cellular immune responses. CsAg17 protein and DNA vaccines increased IFN-γ, IL-2 and IL-12 levels. The Th1 responses characterized with the production of IFN-γ are crucial for protection against diverse infectious pathogens, including *S. japonicum* and *C. sinensis* [20, 28, 29]. IL-2 is another Th1 cytokine that was downregulated in FVB mice susceptible to *C. sinensis* infection [29]. IL-12 stimulates the production of INF-γ and enhances the cytotoxic activity of natural killer (NK) cells and CD8^+^ T lymphocytes [30–32]. It also plays a central role in the activation, differentiation, and expansion of antigen-specific Th1 cells. In CsAg17-vaccinated mice, the increase in the level of IL-12 may enhance the cytotoxicity of NK cells and account for the reduction in *C. sinensis* burden.

Both IL-4 and IL-10 are Th2-associated cytokines and suppress the production of Th1 cytokines such as IFN-γ and IL-2. IL-4 induces the B cell class switching to IgE and is associated with allergy [33]. IL-10 downregulates the production of MHC II molecules, which suppress antigen presentation by CD4^+^ T cells. The production of IL-4 and IL-10 was reported to decrease the protection of mice against *C. sinensis* infection [18, 29]. In contrast, vaccination with CsAg17 protein or DNA was found to suppress the production of Th2 cytokines such as IL-4 and IL-10 but stimulate the expression of Th1 cytokines, which could strengthen the protective effects in FVB mice infected with *C. sinensis*.

Vaccination with CsAg17 protein and DNA minimized the destructive pathological changes in the liver and bile ducts of the FVB mice infected with *C. sinensis*. Taken together, the vaccination with CsAg17 protein and DNA induced an increase in Th1 level and a decrease in Th2 cytokine level, reduced the pathological changes in the bile duct and liver, imparted partial protection, and ameliorated the worm burden *via* cellular and humoral immune responses.

## Conclusions

*Clonorchis sinensis* causes inflammatory injuries to the liver tissue, eventually leading to bile duct cancer. Although an effective drug, praziquantel, is available to eliminate flukes from the bile duct, people are repetitively infected due to their eating habits. Thus, it is imperative to take preventive measures or vaccination to prevent re-infections with *C. sinensis*. Here, both CsAg17 protein and cDNA vaccines induced strong cellular and humoral immune responses in mice. Moreover, in mice challenged with *C. sinensis* metacercariae, vaccination offered protection from liver damage and reduced worm burden. Thus, the *CsAg17* gene and its product are proposed to serve as potent vaccine candidates against *C. sinensis* infections.

## Supplementary information

**Additional file 1: Figure S1.** Purification of recombinant CsAg17 B cell epitope protein. Proteins were separated with 12% gradient polyacrylamide gel electrophoresis (PAGE). Lane 1: uninduced *E. coli* lysate; Lane 2: total lysate of induced *E. coli*; Lane 3: soluble fraction of induced *E. coli* lysate; Lane 4: pass-through fraction; Lane 5; wash-off; Lanes 6, 7: first and second eluates; Lane M: protein molecular weight marker.

**Additional file 2: Figure S2.** Immunoblotting of CsAg17 proteins using mouse anti-CsAg17 B cell epitope immune serum. Lane 1: soluble extract of *C. sinensis* adult; Lane 2: recombinant CsAg17 B cell epitope and normal mouse serum; Lane 3: insoluble recombinant full length CsAg17; Lane 4: recombinant CsAg17 B cell epitope; Lane M: protein molecular weight marker.

**Additional file 3: Figure S3.** Localization of CsAg17 protein in *C. sinensis* adults. Tissues were treated with mouse anti-CsAg17 partial protein immune serum (**a**–**e**), or normal mouse serum (**f**–**j**). *Abbreviations*: SR, seminal receptacle; VS, ventral sucker; Ms, mesenchymal tissue; Egg, intra-uterine egg; In, intestine.

**Additional file 4: Figure S4.** Transcription level of *CsAg17* mRNA during developmental stages. RT-qPCR was performed with the mRNAs obtained from *C. sinensis* metacercariae and adults. Relative transcription level of *CsAg17* mRNA is shown. ***P < 0.001 compared to metacercariae.

## Data Availability

Data supporting the conclusions of this article are included within the article and its additional files. The CsAg17 cDNA sequence was submitted to the GenBank database under the accession number MN381946.

## Abbreviations

ELISA: enzyme-linked immunosorbent assay
IFN-γ: interferon-γ
IL: interleukin
MHC: major histocompatibility complex
Th1: type 1 T helper cell
Th2: type 2 T helper cell
