# Supplementary figures and images for: Clonorchis sinensis secretory protein CsAg17 vaccine induces immune protection
Source: Parasit Vectors. 2020 Apr 25;13:215. doi: 10.1186/s13071-020-04083-5 (PMC7183723; doi:10.1186/s13071-020-04083-5)

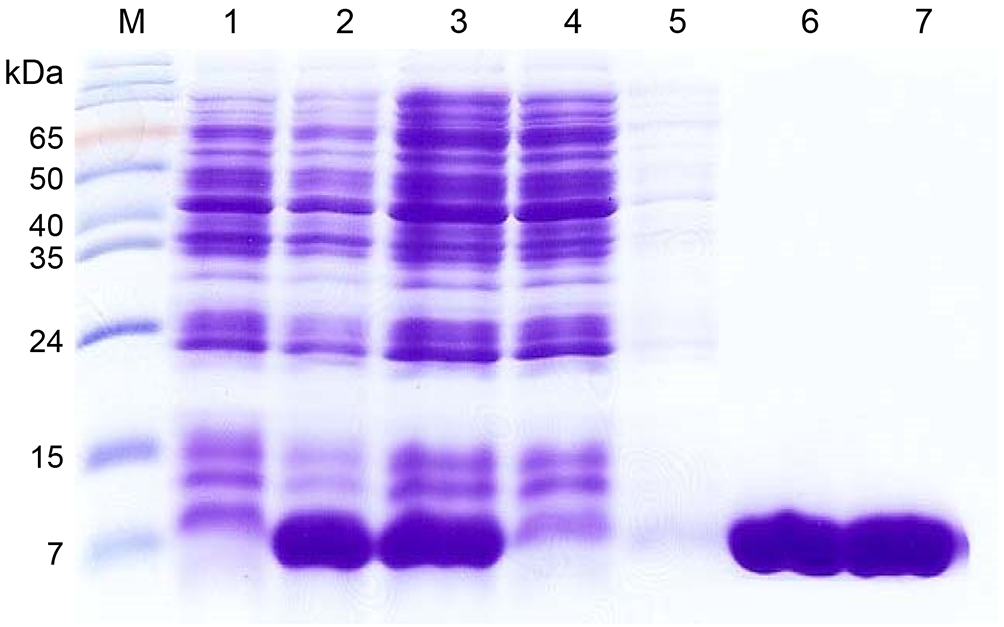

Supplement: Supplementary file 1 — Additional file 1: Figure S1. Purification of recombinant CsAg17 B cell epitope protein. Proteins were separated with 12% gradient polyacrylamide gel electrophoresis (PAGE). Lane 1: uninduced E. coli lysate; Lane 2: total lysate of induced E. coli; Lane 3: soluble fraction of induced E. coli lysate; Lane 4: pass-through fraction; Lane 5; wash-off; Lanes 6, 7: first and second eluates; Lane M: protein molecular weight marker. [file 13071_2020_4083_MOESM1_ESM.tif]

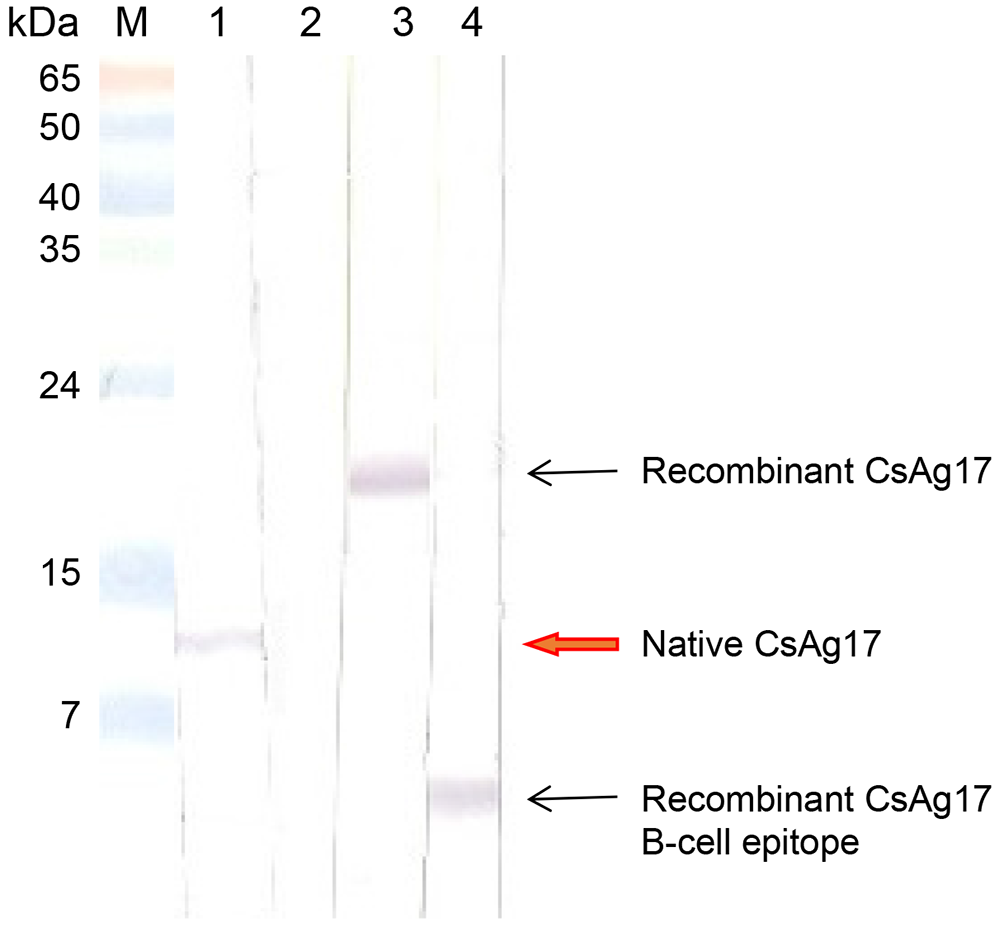

Supplement: Supplementary file 2 — Additional file 2: Figure S2. Immunoblotting of CsAg17 proteins using mouse anti-CsAg17 B cell epitope immune serum. Lane 1: soluble extract of C. sinensis adult; Lane 2: recombinant CsAg17 B cell epitope and normal mouse serum; Lane 3: insoluble recombinant full length CsAg17; Lane 4: recombinant CsAg17 B cell epitope; Lane M: protein molecular weight marker. [file 13071_2020_4083_MOESM2_ESM.tif]

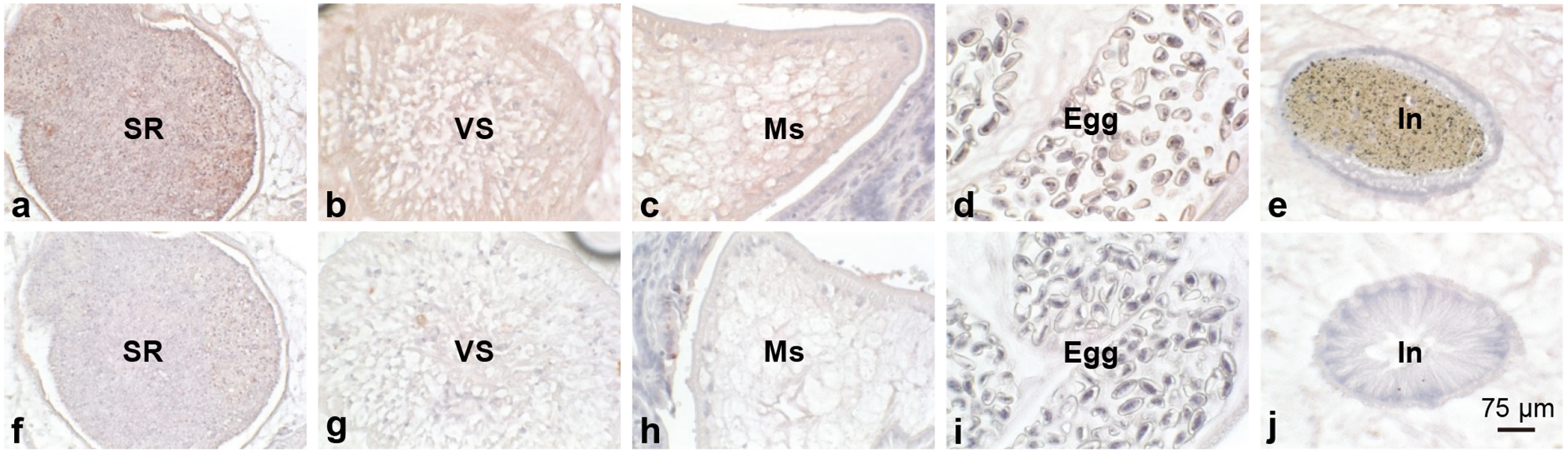

Supplement: Supplementary file 3 — Additional file 3: Figure S3. Localization of CsAg17 protein in C. sinensis adults. Tissues were treated with mouse anti-CsAg17 partial protein immune serum (a–e), or normal mouse serum (f–j). Abbreviations: SR, seminal receptacle; VS, ventral sucker; Ms, mesenchymal tissue; Egg, intra-uterine egg; In, intestine. [file 13071_2020_4083_MOESM3_ESM.tif]

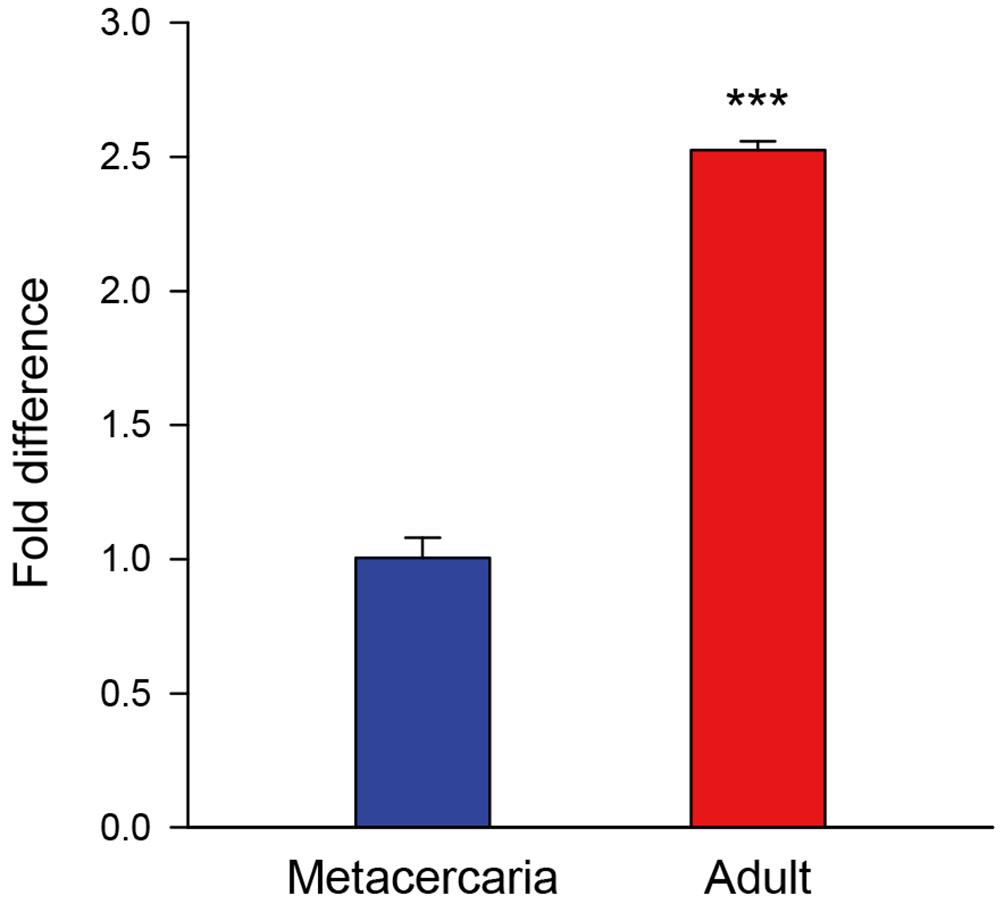

Supplement: Supplementary file 4 — Additional file 4: Figure S4. Transcription level of CsAg17 mRNA during developmental stages. RT-qPCR was performed with the mRNAs obtained from C. sinensis metacercariae and adults. Relative transcription level of CsAg17 mRNA is shown. ***P < 0.001 compared to metacercariae. [file 13071_2020_4083_MOESM4_ESM.tif]
